# 3D Biomimetic Porous Titanium (Ti_6_Al_4_V ELI) Scaffolds for Large Bone Critical Defect Reconstruction: An Experimental Study in Sheep

**DOI:** 10.3390/ani10081389

**Published:** 2020-08-11

**Authors:** Alberto Maria Crovace, Luca Lacitignola, Donato Monopoli Forleo, Francesco Staffieri, Edda Francioso, Antonio Di Meo, José Becerra, Antonio Crovace, Leonor Santos-Ruiz

**Affiliations:** 1 Dottorato di Ricerca in Sanità e Scienze Sperimentali Veterinarie—DMV, University of Perugia, Via S. Costanzo, 4, 06126 Perugia, Italy; alberto.crovace@libero.it (A.M.C.); antonio.dimeo@unipg.it (A.D.M.); 2 Department of Emergencies and Organ Transplantation (DEOT), Strada Provinciale per Casamassima Km.3, 70010 Valenzano (BA), Italy; francesco.staffieri@uniba.it (F.S.); eddagiuseppina.francioso@uniba.it (E.F.); antonio.crovace@uniba.it (A.C.); 3 Departamento de Ingeniería Mecánica, Instituto Tecnológico de Canarias (ITC), Añepa, esq. Tigotán s/n, 35118 Arinaga (Agüimes) Las Palmas de Gran Canaria, Spain; dmonopoli@itccanarias.org; 4 Departamento de Biología Celular, Genética y Fisiología, Facultad de Ciencias, Campus de Teatinos, 29071 Málaga, Spain; becerra@uma.es; 5 Centro Andaluz de Nanomedicina y Biotecnología (BIONAND), Parque Tecnológico de Andalucía, C/Severo Ochoa, 35 29590 Campanillas-Málaga, Spain; 6 Centro de Investigación Biomédica en Red—Bioingeniería, Biomateriales y Nanomedicina (CIBER-BBN), Instituto de Saludo Carlos III, C/ Monforte de Lemos 3-5, Pabellón 11-Planta 0, 28029 Madrid, Spain; 7 Instituto de Investigación Biomédica de Málaga—IBIMA, 29071 Málaga, Spain

**Keywords:** bone, bone fracture, bone repair, metal endo-implant, titanium alloy, additive manufacturing, EBM (electron beam melting), biomechanics, critical-size bone defect, sheep, animal model

## Abstract

**Simple Summary:**

The authors propose a new reconstructive technique that proved to be suitable to reach this purpose through the use of a custom-made biomimetic porous titanium scaffold. An in vivo study was undertaken where a complete critical defect was experimentally created in the diaphysis of the right tibia of twelve sheep and replaced with a five-centimeter porous scaffold of electron beam melting (EBM)-manufactured titanium alloy or a porous hydroxyapatite scaffold. Our results show that EBM-formed titanium devices, if used to repair critical bone defects in a large animal model, can guarantee immediate body weight-bearing, a rapid functional recovery, and a good osseointegration. The porous hydroxyapatite scaffolds proved to be not suitable in this model of large bone defect due to their known poor mechanical properties.

**Abstract:**

The main goal in the treatment of large bone defects is to guarantee a rapid loading of the affected limb. In this paper, the authors proposed a new reconstructive technique that proved to be suitable to reach this purpose through the use of a custom-made biomimetic porous titanium scaffold. An in vivo study was undertaken where a complete critical defect was experimentally created in the diaphysis of the right tibia of twelve sheep and replaced with a five-centimeter porous scaffold of electron beam melting (EBM)-sintered titanium alloy (EBM group *n* = 6) or a porous hydroxyapatite scaffold (CONTROL group, *n* = 6). After surgery, the sheep were allowed to move freely in the barns. The outcome was monitored for up to 12 months by periodical X-ray and clinical examination. All animals in the CONTROL group were euthanized for humane reasons within the first month after surgery due to the onset of plate bending due to mechanical overload. Nine months after surgery, X-ray imaging showed the complete integration of the titanium implant in the tibia diaphysis and remodeling of the periosteal callus, with a well-defined cortical bone. At 12 months, sheep were euthanized, and the tibia were harvested and subjected to histological analysis. This showed bone tissue formations with bone trabeculae bridging titanium trabeculae, evidencing an optimal tissue-metal interaction. Our results show that EBM-sintered titanium devices, if used to repair critical bone defects in a large animal model, can guarantee immediate body weight-bearing, a rapid functional recovery, and a good osseointegration. The porous hydroxyapatite scaffolds proved to be not suitable in this model of large bone defect due to their known poor mechanical properties.

## 1. Introduction

Bone damage due to either pathologies or trauma is a common occurrence in Orthopedics. Osteotomy, followed by bone distraction and auto or allografting, including vascularized bone grafting, are the most currently used therapeutic approaches. Ilizarov, as well as other techniques based on the distraction of membranous bone, rely on the bone self-regenerating potential. The relatively high rate of success provided by these techniques is, however, counterbalanced by the long recovery time, the low compliance by the patient, and the high number of complications [1].

Different biomaterials have been proposed as bone substitutes [2]. Those currently available include 3D ceramic scaffolds, like hydroxyapatite (HA) or β-tricalcium phosphate (β-TCP), and bioactive glasses [3,4,5,6]. However, they are considered to be brittle [7] and easy to fracture, making them unsuitable for bone regeneration in load-bearing scenarios [8,9,10].

There are at least three characteristics that an ideal bone substitute should present: osteoconduction, which is the ability to support bone growth over its surface, osteoinduction, which is the ability to induce, within the surrounding tissues, the differentiation of stem cells into osteoblasts and to promote the formation of new bone by these cells, and osseointegration, the ability to physically and chemically bond to the neighboring bone tissue without intervening fibrous tissue. Several studies have determined that bone substitutes must be porous to allow for the migration of osteogenic cells and vascular ingrowth [11,12].

Several attempts have been made to treat large bone defects with tissue-engineering approaches. Quarto et al. [13] first reported a cell-based tissue-engineering procedure to treat patients with long bone segmental defects, where a 100% hydroxyapatite (HA) porous ceramic was loaded with mesenchymal stem cells. Mastrogiacomo et al. [14] evaluated the performance of a silicon-containing mineral biomaterial (Skelite) in promoting the repair of an extensive, experimentally induced defect in a weight-bearing long bone in a sheep model. Despite the good performances of ceramic/polymeric bone substitutes, several limitations should be considered, like their large variability in biodegradation times and, particularly, their poor mechanical load-bearing ability.

Metallic implants used in orthopedic surgery are numerous and different in structure. Stainless steels were the first reliable metals used as prostheses in orthopedics. The basic elements in steels are iron and carbon and may usually contain chromium, nickel, and molybdenum as additional elements. Compared to titanium implants, stainless steels exhibit a lower strength and corrosion resistance and a greater ductility and stiffness. Their high stiffness makes stainless-steel implants inferior to titanium ones in bone replacement applications [15]. Currently, titanium is the most used metallic biomaterial due to its biocompatibility, resistance to the corrosive activity of the body, and its specific force related to its traction force and rigidity. In the presence of oxygen, titanium gets coated by an oxide layer that increases its biocompatibility [16]. This characteristic makes titanium alloys ideal for bone substitution [17]. Among the different titanium formulations, extra-low interstitial Ti_6_Al_4_V alloy (Ti_6_Al_4_V ELI) is possibly the most common titanium alloy used for implant manufacturing [18,19].

Introducing porosity in metal implants has been proposed as a strategy to reduce its elastic modulus, making it closer to the elastic behavior of bone, thus improving its biological fixation and increasing the longevity of orthopedic implants [20]. In 2007, the process of metal additive manufacturing was introduced in the prostheses industry. This process allows manufacturing three-dimensional metal scaffolds from metal powder, with any desired geometric shape. The computer-designed scaffolds can be faithfully reproduced by a technology in which the titanium powder is melted with either a laser beam (laser beam melting or LBM) or with an electron beam (electron beam melting or EBM). The EBM process was the first to be certified for orthopedic implant manufacturing, because it assures a total fusion of the metal powder, it does not need any postprocess heat treatment, and the mechanical performance of the manufactured pieces are in compliance with ISO:5832-3. EBM-manufactured Ti_6_Al_4_V ELI devices can present different porosity and geometry, two parameters that can be controlled to confer the device with mechanical properties more similar to those of bone. 

The aim of this study was to evaluate in vivo, on a large animal model, the efficacy of highly porous EBM-sintered titanium alloy devices to custom-fit critical-size large bone defects [21,22,23], as compared to porous hydroxyapatite scaffolds. Our hypothesis was that EBM devices, thanks to their biomechanical and osteoconductive properties, would be able to guarantee a more effective repair of the large bone defect compared to hydroxyapatite scaffolds. 

## 2. Materials and Methods

### 2.1. Ethics Statement and Animal Care

After approval by the Italian Ministry of Health (n°425/2018-PR), and in strict accordance with the recommendations in the Guide for the Care and Use of Laboratory Animals of the National Institutes of Health, twelve 3-year-old sheep (weight 45 ± 5.1 Kg) were included in this prospective randomized experimental study. Sheep were randomly divided into two groups: EBM group (*n* = 6) and CONTROL group (*n* = 6) and underwent surgical procedures. All the sheep were subjected to clinical and hematological examinations aiming to assess their health state and to a radiographic examination to verify the complete absence of pathologies in the interested anatomical compartment (i.e., the tibia of the right hind limb).

The animals were placed in their barns at least one month before the start of the study, in order to acclimatize themselves. They had free access to water and were fed fodder and concentrated food.

Sheep were weighed before surgery and at regular intervals during the experimental period. Surgery was performed under aseptic conditions and under both general and spinal anesthesia. All efforts were made to minimize suffering: all sheep were monitored daily in order to detect any alteration of the clinical conditions (food intake and weight loss, urine and feces production, rectal temperature, and behavioral changes).

### 2.2. Description of the Animal Model

A full-thickness 5-cm defect was practiced in the tibial diaphysis of sheep under anesthesia and replaced with a framework of EBM-sintered titanium (EBM group) or porous hydroxyapatite scaffold. The surgical bone defect was considered of critical size (more than twice the diameter of the tibial diaphysis). After surgery, animals were recovered and housed in adequate barns. Follow-ups were performed at 9 and 12 months after surgery with clinical, radiographic (9 and 12 months), and histological (only at 12 months) evaluations.

### 2.3. Titanium Scaffold and Plates

In the EBM group, scaffolds and plates were manufactured by EBM-sintering technology from Ti6Al4V ELI metal powder. Design and manufacturing took place at the Instituto Tecnológico de Canarias (ITC, Agüimes, Spain). The scaffold consisted of a cylinder measuring 40-cm-long × 12-mm-diameter, with high porosity. Indeed, only 10% of the scaffold volume was metal. The modulus of the elasticity of the scaffolds was 1 GPa, which is between those of the cancellous and the laminar bone. The scaffold design also took into account a deformation under 2% for the pores in load conditions. The mean pore size was 1.5 × 2.4 mm.

**Figure 1:**
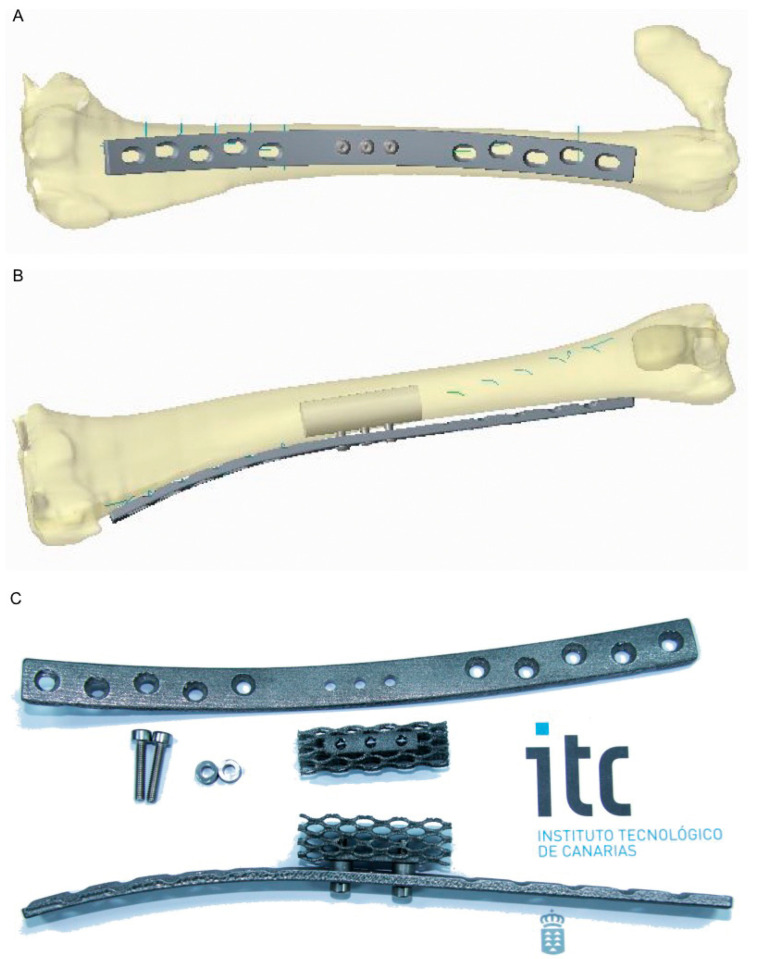
Computer-assisted design of the scaffold and osteosynthesis plate. (**A**) Frontal view, showing the plate. (**B**) Lateral view, showing the position where the scaffold should fit after the resection and how the osteosynthesis plate shape fits the tibial profile. (**C**) Electron beam melting (EBM)-sintered Ti_6_Al_4_V ELI scaffold, osteosynthesis plate, and screws.

Osteosynthesis plates were custom-designed from *dicom* files obtained through a CT study performed in advance to the tibia of the sheep used in the study. The goal of customization was to be able to adapt the metal plates to the medial profile of the tibia without intraoperative modification (Figure 1A,B). They consisted of neutralizing low-contact plates with holes to fit the screws that would anchor to the bone and two smaller holes where smaller screws anchored to the metal scaffold (Figure 1).

Porous metal scaffolds and plates were designed on a CAD model (computer-aided design) and manufactured by metal additive manufacturing machines (laser sintering and electron beam melting). The geometry used for our experiments was a gyroid type, which was modeled by CAD in an isotropic shape for critic and load-bearing experiments at a defined rate of metal on a defined volume of a porous structure. Under isotropic conditions, and at the same porous rate (in the range around 90%), gyroid-type structures present 25% more resistance than the more common and used diamond structures.

Our gyroid structures have been stretched up to 50% along the load direction, increasing the load resistance to the double.

Scaffolds and plates were produced by additive manufacturing through electron beam melting technology (ARCAM B12). For this purpose, Ti_6_Al_4_V–ELI powder with 45–70-µm granulometry was deposited in fine layers (70-µm-thick) and selectively fused, slice by slice, with a high-power electron beam that generates the energy needed for a melting spot. The process took place in vacuum (pressure of 1 × 10^−5^ mbar or better throughout the entire build cycle) and at high temperature, resulting in stress-relieved components with material properties better than cast and comparable to wrought materials. During the melting process, a partial pressure of He (2 × 10^−3^ mbar) was introduced.

The cleaning process took place by sandblasting with particles of the same Ti_6_Al_4_V–ELI powder, with a granulometry around 50–60 µm. Afterwards, the pieces were further cleaned in an ultrasound bath with lightly soapy distilled water for 15 min, followed by a last ultrasound bath of 15 min in pure distilled water [24]. The scaffold was autoclaved at 134 °C for 10 min before clinical application.

### 2.4. Porous Ceramic Scaffold

In the CONTROL group, the scaffold employed was made of silicon-stabilized tricalcium phosphate (Si-TCP); the silicon, which substitutes for phosphorus, is distributed throughout the material’s crystal structure and provides a multiphase composition, consisting of approximately 67% Si-TCP and 33% HA/-TCP. The scaffolds used in this study were cylinders with a central canal (18 mm, OD 6 mm, ID 50 mm) and had a porosity level of 60%, with a pore size between 200 and 500 µm.

### 2.5. Surgical Procedure

Sheep underwent fasting for 12–24 h prior to the start of anesthesia and were given antibiotic therapy (20 mg/kg ampicillin), as well as anti-inflammatory/analgesic therapy (1mg/kg ketoprofen) one hour prior to surgery.

After positioning of an intravenous catheter (20 G) into the auricular vein, animals were sedated with Diazepam (0.4 mg/kg). Fluid therapy was performed with Ringer’s lactate (10 mL/Kg/h).

After an adequate level of sedation was achieved, sheep were placed in right lateral recumbency in order to perform spinal anesthesia at the level of the L6–S1 intervertebral space. After the aseptic preparation of the lumbar area, a mixture of 2% lidocaine (1 mL/10 kg) and buprenorphine (10 mcg/kg) was injected into the subarachnoid space with a spinal needle. The adequate onset of the spinal block was confirmed by the loss of the muscular tone (relaxed anus sphincter and rear limb muscles) and by clamping the skin on the area of surgery in order to confirm the sensory loss. 

**Figure 2:**
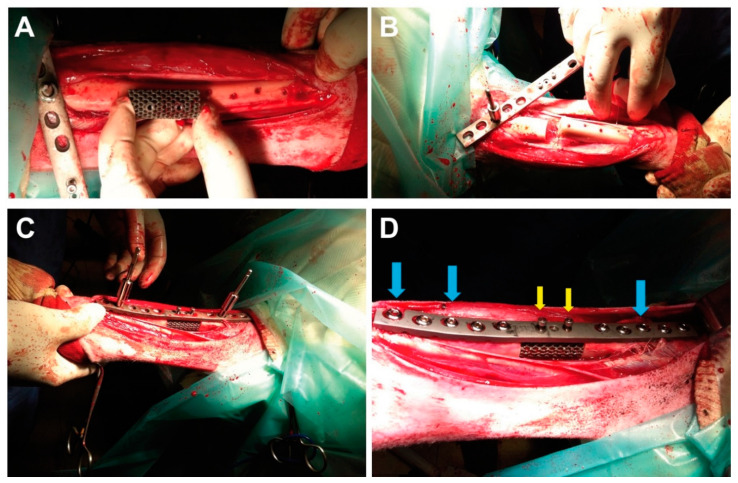
Photographs illustrating the surgical implantation of a titanium scaffold (experimental group). (**A**) The tibia has been exposed, and the scaffold is shown next to it. (**B**) A five-centimeter full-thickness defect has been practiced. (**C**) The scaffold has been placed on the defect, and the tibia is being stabilized with a custom-made osteosynthesis plate. (**D**) Final result, showing the scaffold and osteosynthesis plate, which is held in place by screws that anchor in the bone (blue arrows) or in the scaffold itself (yellow arrows). In the CONTROL group, the surgical procedure was similar, but the defect was filled with hydroxyapatite.

Sheep were then positioned in left lateral recumbency. After scrubbing, the right limb was covered with a stockinet and towels and a long incision from the medial condyle of the tibia to the medial malleolus was made on the skin with a n° 23 surgical blade. Afterward, the subcutaneous tissue was incised on the same line; the tibial muscle was pulled away with the aid of an autostatic retractor to avoid accidental soft tissue injury. After bleeding control, the periosteum of the tibia was elevated from the bone and removed in the area where the scaffold was going to be placed. A five-centimeter-long ostectomy was performed in the diaphysis of the right tibia with an oscillating saw and replaced, in the EBM group, with the EBM-sintered macroporous titanium scaffold (Figure 2A,C,D) and, in the CONTROL group, with a porous hydroxyapatite scaffold. In the EBM group, the tibia was stabilized with a custom-made, EBM-sintered, titanium neutralizing plate and 10 cortical screws of 4.5-mm-diameters, five of them located proximally and five placed distally (Figure 2C,D). The scaffold was fixed to the custom-made plate with two threaded screws of 3 mm of diameter and inserted in preshaped holes located in the plate and the scaffold (Figure 2C,D). 

In the CONTROL group, the tibia was stabilized with a neutralizing titanium plate of the same length and eight holes and screws, compared to the EBM group. The surgeons verified that the scaffold was perfectly adherent to the proximal and distal borders of the bone and that its section had a smaller diameter than the bone contacting it on both its proximal and distal sides. The sutures of the muscles and subcutaneous tissue and skin covered the plate, while the periosteum was incompletely sutured because of the plate width; the scaffold was covered only with the sutures of muscle and subcutaneous tissue and skin. The wound was covered with a sponge and fixed with adhesive bandaging. Sheep were recovered from anesthesia and were allowed to move freely in the barn. 

For five days after surgery, sheep received antibiotics (benzylpenicillin/streptomycin every 12 h) and anti-inflammatory drugs (ketoprofen 1 mg/kg IM every 12 h). After 10 days, the skin sutures were removed. Nine months after implantation surgery, the osteosynthesis plates were removed under general anesthesia. Sheep were euthanized 12 months after surgery, and the tibiae with the implants were harvested and processed for histological analysis.

### 2.6. Noninvasive Follow-Up

The outcome was noninvasively followed up for up to 12 months by monthly X-ray acquisitions. Clinical examinations included observation of the gait and the evaluation of the treated tibia to assess the limb functional and anatomical status. 

### 2.7. Histological Analysis

Dissected tibiae were fixed by immersion in 10% neutral-buffered formalin (4% formaldehyde) for 7 days. The tibias were cut into three segments, and each of them was divided into 4-mm-thick slices with an EXAKT 300 CP bandsaw and post-fixed in the same fixative for another 4 days. They were then dehydrated by immersion in a graded ethanol series and embedded in Technovit 7210 VLC resin, which was lightly polymerized. After polymerization, resin blocks were sectioned into 0.4-mm-thick slides with a diamond saw and polished with an EXAKT 400 GRINDING SYSTEM until obtaining 50-µm-thick histological sections. Sections were stained with von Kossa staining to reveal calcified bone, as described elsewhere [25]. Briefly, the sections were incubated in 1% silver nitrate aqueous solution under ultraviolet light for 20 min and rinsed thoroughly five times in bi-distilled water. Unreacted silver was removed by washing with 5% sodium thiosulfate aqueous solution, followed by new washes in bi-distilled water. Nuclei were counterstained by immersing the section for five minutes in Nuclear Fast Red Solution (Sigma-Aldrich, St. Louis, MO, USA).

## 3. Results

### 3.1. X-ray and Clinical Follow-Up

**Figure 3:**
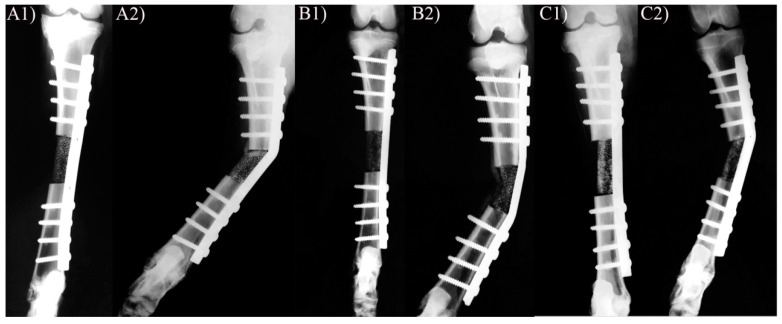
X-ray of three sheep tibiae representative of the CONTROL group 20 days after surgery. For each animal, two images are shown: the one on the left corresponds to a frontal plane in the immediately postoperative CONTROL (**A1**,**B1**,**C1**) and the one the right to an x-ray taken after 20 days (**A2**,**B2**,**C3**). Note the failure of the implant, with bending of the plate and medial deviation of the tibia.

All sheep in the postoperative X-rays showed a correct position of the plate and full contact between the scaffold’s ends and the adjacent bone stumps. All the sheep started to walk, using the treated limb, immediately after recovery from anesthesia. All sheep in the CONTROL group showed moderate/severe lameness on the treated leg and signs of general discomfort in the immediate post-operative time. After 20 days, gait did not improve, and lateral deviation of the tibial axis could be appreciated. X-ray imaging confirmed the failure of the implants, bending of the plates, and limb deviation (Figure 3). For clinical and ethical reasons, all the animals in the CONTROL group were euthanized, and the group was removed from the experimental design.

**Figure 4:**
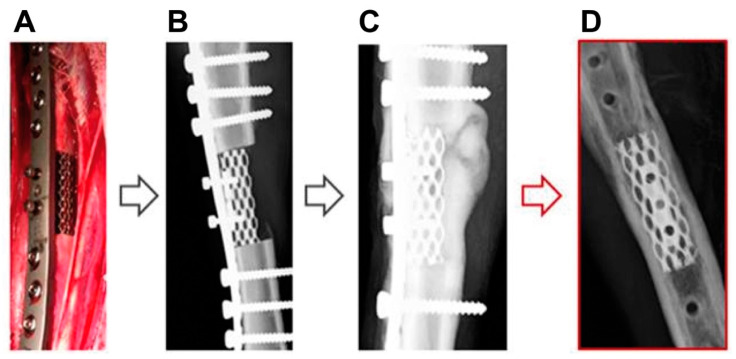
(**A**) Photograph of the operation field in an animal of the experimental group, showing the tibia and the 5-cm-long macroporous scaffold (asterisk). The osteosynthesis plate is anchored with screws to both the bone (big arrows) and the scaffold (small arrows). (**B**–**D**) X-ray images of the sheep-operated tibiae taken right after surgery (**B**), 2 months after surgery (**C**), and 9 months after surgery (**D**).

After two months, clinical examination of the EBM group showed normal gaits on the treated limbs with no lameness, being the articular movements of knee and tarsus normal, without modification of the range of motion. The sheep showed no signs of pain upon articular movements and tibia manipulations during. The clinical examination showed no implant dislocation or screw failures and no signs of infection. The X-rays acquired at this moment did not show adverse reactions around the scaffold or around the screws. The scaffolds were stable and in contact with the bone interfaces. An osseous callus (periosteal new bone formation) was visible around the whole implant length, particularly at the lateral side of the tibia. At the interface between the bone ends and the scaffold ends, bone ingrowth into the scaffold was evident (Figure 4C). 

The monthly examinations showed that the sheep were progressively increasing the load on the operated limb during their walks. Nine months after surgery, complete functionality of the operated limbs was apparent, and no anatomical anomalies were detected during palpation. X-rays confirmed the stability of the implants and their integration within the host bone. A layer of cortical bone was visible around the implant that was continued with the cortical layer of the adjacent bone. Within the scaffolds, a radio-dense aspect suggested bone ingrowth into the pores of the scaffold. (Figure 4D). On the basis of these X-ray images, it was decided to remove the custom-made osteosynthesis plate and all the screws that anchored it to both the bone and the scaffold. After the implant’s removal, the sheep did not show any signs of functional deterioration of the treated limbs.

Three months after removal of the titanium plates, the sheep were euthanized and the tibiae harvested for histological examination.

### 3.2. Histological Evaluation

**Figure 5:**
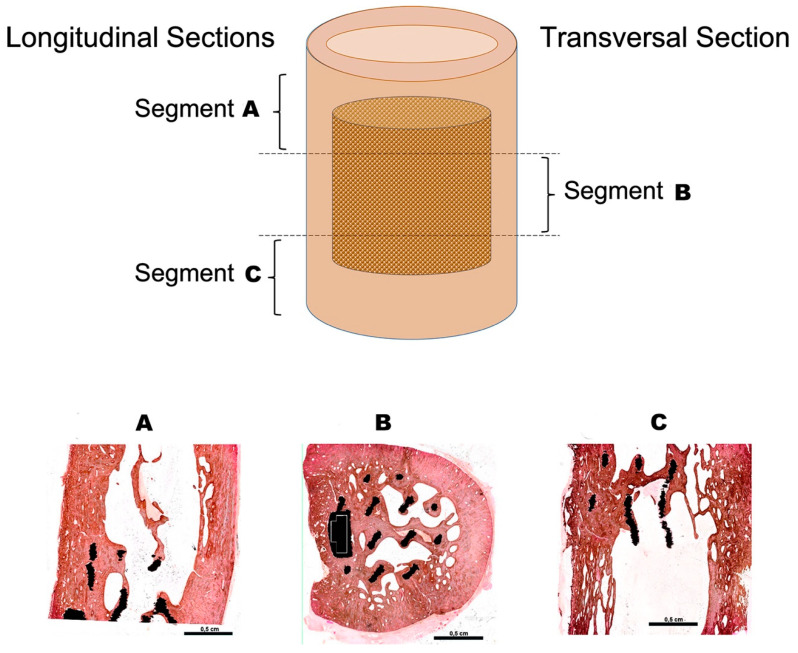
Histological processing of the sheep tibiae from the experimental group. Upper panel: Each bone, containing the implant, was sectioned into three segments. Longitudinal sections were obtained from the ends (segments **A** and **C**), while the central part was cut transversally (segment **B**). Lower panel: Panoramic overview of the von Kossa-stained methyl-methacrylate sections. (**A**,**C**) are longitudinal sections. (**B**) is a transversal section. The bone is stained in brown, and the titanium of the scaffold appears black in color. Note how the bone trabeculae are connecting the metal bars that compose the porous titanium scaffold.

As seen in both longitudinal and transversal sections (Figure 5), the titanium implant was correctly positioned, centered within the medullary canal. No signs of chronic inflammation or foreign body reactions were observed.

A newly grown cortical bone surrounded the scaffold, being this new cortical bone continuous with that of the adjacent bone ends (Figure 5A–C). The bone tissue was deposited adjacent to all titanium trabeculae in all the length of the implant (Figure 5A–C). 

**Figure 6:**
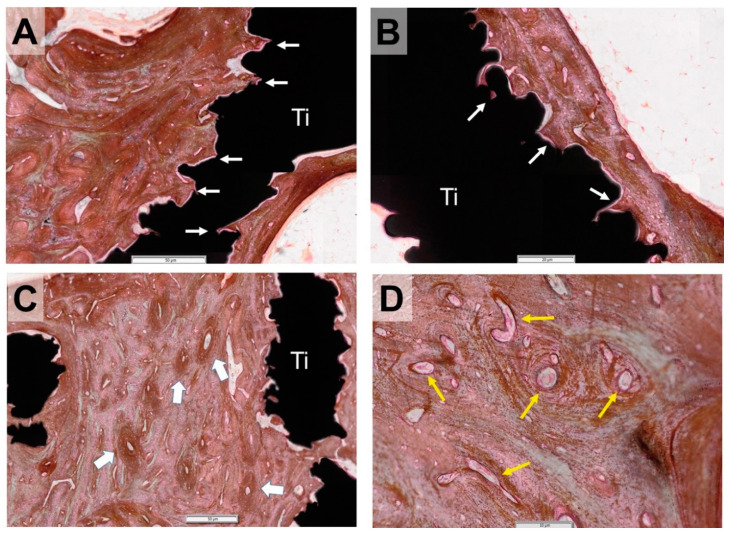
Details of von Kossa-stained bone sections showing the bone scaffold interface. (**A**,**B**) The bone is deposited directly on the titanium (Ti), with no fibrillar tissue in between. The bone fills all the little cavities and interstices of the scaffold surface (arrows). (**C**) The bone remodeling is appreciable by the occurrence of new osteons overlapping the previously formed bone (thick arrows). (**D**) Numerous blood vessels are visible in the newly formed bone (yellow arrows). Bars: 50 µm in (**A**,**C**), 20 µm in (**B**), and 10 µm in (**D**).

There was direct contact between this newly formed bone and the titanium trabeculae, with no soft or fibrillar tissue between the metal and the bone. Indeed, the bone tissue profile closely matched that of the titanium (Figure 6A,D) due to bone growth in all the interstices of the titanium surface.

Newly grown bone trabeculae not only surrounded the metal bars that conformed the titanium scaffold, they also bridged them, creating a network of interconnected bone and titanium trabeculae (Figure 5A–C). This suggests that the scaffold has offered the bone tissue a surface to grow atop, and, on colonizing the scaffold, the bone has been guided from one stump to the other, resulting in the full regeneration of the excised critical-size defect. In other words, osteoprogenitor cells found in the EBM-sintered titanium scaffold a surface suitable for adhesion, colonization, differentiation, and the production of a new bone matrix. 

Numerous blood vessels were visible in the calcified matrix (Figure 6D), indicating bone vitality. Osteons overlapped each other, denoting bone-remodeling processes (Figure 6C).

## 4. Discussion

The results of this study showed that in a large animal critical bone defect, EBM scaffolds are able to guarantee a rapid loading of the animal and an excellent anatomical repair of the bone defect. On the contrary, porous hydroxyapatite scaffolds proved to be unsuitable for this experimental model.

The reconstruction of large bone defects has traditionally been achieved by the use of resorbable bone substitutes [14,26] or permanent metallic prosthetic implants [15,18,19]. Unlike bone substitutes, which cannot hold the load bearing, metal prosthetic implants allow early functional recovery. Postoperative success in the mid- and long-term depends on two critical factors: load-bearing capacity and osseous integration. Our results showed that traditional metal implants fail in the early stage of the treatment because of the inability to ensure the load bearing. Indeed, critical defects can lead to failure if adequate implant support techniques are not used, such as an orthogonal second plate, external fixators, blocked nails, or bandages or suspensions, and, in any case, subtraction from the load [21,23,26,27,28,29]. This aspect was empathized in our large animal model, which was aimed to stress also the aspect of the load bearing. Hydroxyapatite is known to be osteoconductive [30], but in our case, we think that the failure of the implant was related to the poor load-bearing ability of the scaffold. Moreover, previous publications proved also that traditional implants tend to fail also in the medium and long-term due to poor osteointegration—because they are solid, bones cannot grow inside them, and a structural connection between the metal and bone is not formed. Some manufacturers have incorporated porous surfaces in some elements of their prostheses in order to amend this problem [31].

An ambitious regenerative approach was undertaken in this work: a metal scaffold was designed with a 90% porosity and only 10% metal. The rational of the design was to provide the scaffold with enough strength to assume load bearing but with an elastic modulus closer to that of bone and a high porosity to allow bone ingrowth inside the implant. The results obtained in this study proved that EBM devices were able to guarantee the complete repair of a large bone defect, achieving both mechanical strength and osseointegration.

Although, in our study, we employed a titanium scaffold consisting of a gyroid architecture with porous size of 1.5 × 2.4 mm with an elongation of 2% along the mechanical load, another study of smaller porous sizes (400–700 µm) was recently implanted in dogs’ tibiae, providing optimal osteointegration. [32]

Our results show that functional recovery was almost immediate, as sheep could walk on their four legs after recovery from the surgical intervention. X-rays showed that the bone grew until totally bridging the gap, and histology showed that the bone tissue had grew in apposition to the metal trabeculae and, also, built bony bridges in between them, suggesting that the implanted scaffold not only supported but, somehow, guided the bone growth, suggesting also that the load bearing was assumed by both the scaffold and the bone.

Several technical considerations were incorporated into the surgical procedure for placing the scaffold: the scaffold diameter was slightly smaller than that of the bone to allow cortical bone growth, and compression at the scaffold-bone interface was applied (the scaffold was press-fit) to guarantee the contact between the implant and the bone tissue. However, in this model, we did not consider the potential impact of using a scaffold of a smaller diameter on the load transmission and mechanical stress at the level of the scaffold-bone interface. Further studies are required to evaluate this specific issue. 

To obtain a rapid load bearing, a rigid osteosynthesis plate was used that would support the load until the scaffold became integrated and stable. This plate was a critical part of the device, as an inadequate fixation could displace the scaffold. Our use of a custom-made titanium osteosynthesis plate guaranteed the perfect modeling of the plate to the bone, without the stress and uncertainty associated with manual modeling at the theater. 

The stiffness of the fixation has an enormous influence on the healing process in which ossification occurs [33]. In fact, interfragmentary micromotion during flexible fixation can stimulate callus formation, improving the healing process, whereas unstable fixation can prevent the repair process [34,35].

The choice of hydroxyapatite scaffolds was based on our previous experiences and considering also the literature. Indeed, these scaffolds proved to be adequate in previous studies. However, as the reviewer noticed, in our model, we used a plate to fix the ostectomy and the scaffold, rather than an external fixator, as previously reported. The reason for choosing a different fixation technique was based on the need to produce a custom-made implant [26].

The other critical factor for implant success was the shape and geometry of the scaffold, which were meant to mimic the characteristics of the metal scaffold to those of bone in terms of porosity, resistance, and elasticity.

The efficacy of the EBM devices was clearly proved in our study, in which, in the CONTROL group, the combination of a standard titanium plate with an hydroxyapatite scaffold was not able to guarantee the load bearing in the first days after surgery, causing the failure of the implants in all animals.

Histological images showed that the healing of the tibial bone resulted in the formation of a new cortical bone that bridged the 5-cm-wide defect created by osteotomy. This cortical bone was continued with a trabecular meshwork of lamellar bone that had been deposited in apposition to the titanium trabeculae of the implant. The formation of lamellar bone adjacent to the titanium trabeculae, in close contact with the metal surface, proved the excellent osteoconductivity of the EBM-sintered titanium alloy.

We can suppose that the natural healing capacity of the bone was influenced by the biomechanical properties of the implants. While the implants used in the CONTROL group took the entire mechanical load, thus preventing bone growth (and indeed, causing bone recession), the EBM-sintered titanium implant might have not taken the entire load. Indeed, it may have played a double role: on one side, it offered an osteoconductive surface where new bone layers could be deposited, so guiding the bone to heal and bridging the defect; on the other side, as new bone layers were deposited by apposition next to the titanium trabeculae, these metal trabeculae could have transmitted to the bone tissue the mechanical load that they were holding. By doing so, the metal trabeculae were possibly transmitting a biomechanical stimulus to the bone tissue that enhanced its healing response. This idea is supported also by several studies that have shown that mechanical loading is essential for maintaining and regulating bone mass [36,37].

The direction of loading has also been shown to influence fracture repair, too. Thus, moderate axial loading of the callus stimulates bone formation [38]. The osteoblastic response depends on the type, duration, and magnitude of the strain [39,40]. Several in vivo and in vitro studies demonstrated that bone cells respond to the physical stimulus with biochemical activities regulating proliferation and differentiation [41,42]. Recently, it is believed that osteocytes, which have an extensive network of cellular filopodia processes that connect and communicate with osteoblasts and osteoclasts, would sense and translate them into biochemical signals and then transmit them to osteoblasts and osteoclasts on the bone surface to regulate bone formation and resorption, respectively, in response to mechanical stimulation [43]. Other authors confirmed with their study on sclerostin that Sost downregulation is an obligatory step for mechanotransduction. The mechanical stimulation reduces sclerostin expression, suggesting that osteocytes might coordinate the osteogenic response to mechanical force by locally unleashing Wnt signaling, and they have demonstrated that cortical bone areas exposed to high mechanical strain exhibit a reduction in sclerostin-positive osteocytes that are associated with higher bone formation on adjacent periosteal surfaces [44,45,46,47]. These events might have occurred in our model, according to our results, in terms of periosteal production on the external side in respect to the medial side, contacting the plate. Considering that, in the CONTROL group, we had 100% failure of the implant in the early postoperative period, compared to the EBM group. We may assume that, in our animal model, part of the mechanical load was applied directly to the scaffold, which played a critical role in the sustainability of the load. This made the bone “work”, stimulating its healing. After the plate was removed, a new load distribution enhanced the bone remodeling. For this reason, the lamellar bone initially placed next to the titanium presented signs of active remodeling, with new osteons overlapping the old ones. 

Recently, another paper has been published about the use of a scaffold made of Ti_6_Al_4_V for a study on metatarsal bone defects in goats [48]. These authors concluded that porous Ti_6_Al_4_V alloy scaffolds fabricated by EBM can be tailored to have better cytocompatibility and mechanical-adapted properties for repairing large segmental bone defects. Our results agree with their study. According to their and our results, scaffolds can be designed with matched morphology and mechanical-adapted properties to provide early load-bearing for large segmental bone defects, while acquiring favorable bone ingrowth in large porosity, which favors osseointegration in the long term. 

## 5. Conclusions

In conclusion, we have shown evidence of the potential of additive-manufactured porous Ti_6_Al_4_V scaffolds as bone defect-repairing devices.
